# Estimation of genetic parameters for reproductive traits in Korean dairy cattle

**DOI:** 10.5713/ab.24.0455

**Published:** 2024-10-25

**Authors:** Jae-Gu Lee, Jeongwoo Seo, Mahboob Alam, Hyungjun Song, Seokhyun Lee, Joohyeon Cho, Chang-Gwon Dang, Joonho Lee

**Affiliations:** 1 Animal Breeding and Genetics Division, National Institute of Animal Science, Rural Development Administration, Cheonan 31000, Korea; 2 GeneApps Co., Ltd., Seoul 06105, Korea; 3 Current affiliation: Department of Agricultural Biotechnology and Research Institute of Agriculture and Life Sciences, Seoul National University, Seoul 08826, Korea; 4 Dairy Cattle Improvement Center of NH-Agri Business Group, National Agricultural Cooperative Federation, Goyang 10292, Korea

**Keywords:** Genetic Correlation, Heritability, Korean Holstein Cattle, Multiple Lactation Animal Model, Reproductive Traits

## Abstract

**Objective:**

In Korea, dairy cattle breeding programs have historically prioritized productive, conformation traits, leading to positive improvements, yet reproductive traits have lagged in development. This study was conducted to develop the breeding program of key reproductive traits in the Korean dairy cattle population.

**Methods:**

Utilizing data from 7,596 farms and over seven million observations, we conducted quality control to rectify manual entry errors and selected traits in line with international genetic evaluation standards. Traits analyzed included heifer conception rate (HCR), interval from calving to first insemination (CF), cow conception rate (CCR), interval from first to last insemination (FL), and days open (DO). Genetic parameters were estimated using a single trait animal model for HCR and a multiple lactation animal model for CF, CCR, FL, and DO, considering contemporary group of herd-insemination year, insemination month, and monthly age as fixed effects.

**Results:**

Results showed low heritability estimates, ranging from 0.007 to 0.035 across different traits and lactations. Theoretical reliability appears to be low on average due to the influence of heritability, but it showed sufficiently high reliability in some sires (over 0.8). In terms of genetic and phenotypic trends, capacity for reproductive traits declined for a long time until around 2014. In recent individuals, improved trend can be found.

**Conclusion:**

This study addressed the critical need for enhancing reproductive efficiency to complement the existing breeding goals, thereby supporting sustained economic viability in the dairy industry. The results underscore the need for improved data quality and methodological adjustments for reproduction records to enhance the genetic evaluation of dairy cattle in Korea.

## INTRODUCTION

The dairy cattle breeding programs in Korea have mainly focused on improving productive traits in the past, resulting in favorable outcomes [1–3]. Subsequently, conformation traits have been introduced into the breeding program and more recently, longevity traits have also incorporated [4]. Aft the same time, reproductive traits have received less interests resulting less direct improvement. However, the demand for improving reproductive traits is growing significantly due to the enhancement of major economic traits and the integration of additional economic traits into Korean dairy breeding objectives [5,6]. Reproductive traits in dairy cattle not only influence offspring production but also impact the efficiency of milk yield since all cows can produce milk after calving [7–9]. Some reproduction efficiency traits, such as the conception rate, could affect how effectively the target cows exist in the milking state and imply that reproductive traits still hold sufficient economic value despite their less economic importance than production traits [10].

Numerous research results indicate the difficulty of improving reproductive traits [11]. The main challenge lies in the low heritability of these traits as well as limitations in data collection and quality which further complicates the improvement of reproductive traits [12]. Also, most reproductive traits have atypical distribution characteristics of phenotypic data.

Besides, reports also suggest antagonistic relationships between productive and reproductive traits [13–15]. This implies that the genetic capacity for reproductive ability in Korean dairy populations, which historically focused on improving productive traits, may be relatively lower. Consequently, the urgency for enhancing the reproductive ability of the Korean Holsteins become even more profound.

Although an official nationwide genetic evaluation for reproductive traits has yet to be made in Korea, preparatory steps are taken to collect preliminary data on reproductive traits with a target for the future development breeding systems regarding fertility traits. This study aims to evaluate the genetic ability of reproductive traits in Korean Holstein populations by estimating the genetic parameters of selected traits. For this purpose, insemination records and calving records were collected and processed, and an appropriate model for genetic evaluation was selected.

## MATERIALS AND METHODS

### Animals, phenotype, and pedigree data

In this study, insemination and calving records from 7,596 farms spanning from 2000 to 2022 were collected from the Nonghyup Dairy Cattle Improvement Center (DCIC), Korea. The raw dataset encompassed 7,043,078 observations over 30 columns. Most individuals had multiple records, often containing one or more insemination entries per parity. The number of heifers with insemination and calving records was 688,345, while cows accounted for 1,096,695. Additionally, pedigree data for 1,880,573 animals were collected from the Korea Animal Improvement Association (KAIA, Seoul, Korea) and utilized to trace the ancestry of cows with records related to reproductive traits.

### Quality control

The original dataset contained potential inaccuracies, as farm owners or their on-site staff manually entered many insemination records. To improve the data quality, we excluded outliers with erroneous date details, incorrect identification, heifer ages below 250 and above 450 days, and cow ages under 450 days. Accurately determining the date of successful conception was crucial, as this data underpins various trait analyses. Solely relying on the most recent insemination date as evidence of successful conception is problematic; there were instances where redundant inseminations for cows already conceived were documented, or some inter-insemination records were omitted in specific farms. To mitigate these issues, we excluded records with gestation lengths (GL) shorter than 260 days or longer than 300 days. We considered the insemination record with a GL closest to the typical 280 days as indicative of a successful pregnancy. Records with a GL exceeding this standard gestation period suggested the absence of the insemination record leading to a successful pregnancy, whereas a shorter GL indicated post-conception insemination entries, likely due to managerial errors. We disregarded such entries, deducing them to be more representative of human errors than genuine reproductive performance.

Another consideration was the exclusion of cases where all individuals on a particular farm had only one insemination record. Such cases could arise from farms possessing cows of exceptional reproductive efficiency that conceive after just one insemination, farms with a small breeding scale, or farms that record only successful inseminations for reasons not explicitly stated. Even though excluding these data points carries a risk of removing valid observations, they were removed to mitigate potential recording errors and enhance the accuracy of further estimation.

### Trait selection and data processing

Identifying and utilizing the appropriate traits for enhancing cows’ reproductive ability is essential. Currently, the reproductive abilities are absent in Korean diary breeding objectives. Therefore, referencing the trait selection from advanced countries that progressed in this domain could offer a more efficient strategy. Furthermore, preemptively selecting traits that align with specific categories would facilitate their integration into international genetic evaluations. Female fertility traits used in this Interbull pilot evaluation were classified as 5 traits [16,17]. Trait 1 (T1) represents the maiden heifer’s ability to conceive. Trait 2 (T2) signifies the lactating cow’s ability to recycle post-calving. Trait 3 (T3) and Trait 4 (T4) demote a lactating cow’s ability to conceive and a relative measurement, respectively. Trait 5 (T5) captures lactating cow’s measurements of interval traits calving-conception. For this study, T1 was redefined as the heifer conception rate (HCR), calculated as the reciprocal of the number of inseminations. The T2 represented the interval from calving to the first insemination (CF). The cow conception rate (CCR) and the interval from the first to the last insemination (FL) indicated the T3 and T4, respectively. The term days open (DO), which was calculated as the difference between the dates of successful insemination and previous calving, was used to indicate the T5. In alignment with the five categories of the International Genetic Evaluation for reproductive traits, certain data restrictions were applied during trait selection. For calculation of the conception rate, entries with 12 or more inseminations were deemed outliers. The interval for CF was set between 20 and 230 days. Any FL greater than 365 days was also categorized as an outlier. These outlier criteria for CF and FL closely align with Muuttoranta et al [18]. For CCR, a maximum of 11 inseminations were permitted in this study, in contrast to the 10 allowed in their study.

### Modeling and estimation of genetic parameters

For the trait derived from heifer data (HCR), a single trait animal model was used due to the absence of repeated parity data. Meanwhile, the other four traits utilized a three-lactation animal model, treating them as distinct traits, given the consistent influencing factors. The reproductive traits are influenced by several factors: herd, insemination year, insemination season, and age at insemination. To ensure the fixed factors were estimable and considering connectedness, we evaluated two types of contemporary groups: herd-insemination year-season (HYS) and herd-insemination year (HY) combined with another term, insemination month (IM). After comparing the estimation results and data utilization, the HY contemporary group combined with IM was selected. The data used for the HY contemporary group are based on the year of the first insemination date in a particular parity (lactation). Even if there are multiple inseminations, only the information from the first insemination date was used. Therefore, even if there are many inseminations within a parity and the insemination dates extend into the following year, the data will only reflect the year of the first insemination. For the insemination age factor treated as a fixed effect, two types of age groupings were considered. The first type categorizes animals as young, normal, and old, based on quartile distinctions (with ages falling within the interquartile range classified as ‘normal’). The second type uses monthly age (MA), ranging from 9 to 25 for heifers, 18 to 42 for the first parity, 30 to 54 for the second parity, and 42 to 66 for the third parity. Upon further evaluation of estimation results and data utilization, the age grouping using MA was selected. In other studies, the inclusion of the service sire as a random effect has been suggested [19]. However, due to missing records and the presence of errors in our dataset, this approach was not feasible in the current study. The models used to estimate the genetic parameters are outlined as follows:

For trait T1: T1 = HY+IM+MA+*u*+*e*
  (Single trait animal model)
For trait T2 to T5: T2 = HY+IM+MA+*u*+*e*
  (Multiple lactation animal model; the models for T3, T4, and T5 are identical to this)

where, T1 represents HCR. T2 to T5 correspond to CF, CCR, FL, and DO for the first through third lactations, respectively. HY denotes the herd-insemination year contemporary group in each lactation. IM stands for the insemination month. The fixed effect of monthly age, MA, is also incorporated in the models. Regarding random effects, *u* is the animal genetic effect, while *e* represents the residual.

To accurately estimate the genetic correlation among the lactations for T2 to T5 using the multiple lactation model, the dataset included only individuals possessing records for all three lactations. Furthermore, the HY contemporary group was restricted to levels of 3 or higher across all three lactations.

The genetic parameters (heritability, and genetic correlation) for three lactations traits were estimated using BLUPF90 software [20].

The theoretical reliability for the estimated breeding value was calculated as follows using PEV and the inbreeding coefficient [21].


$$Reliability=1-\frac{PEV}{\sigma_{g}^{2}(1+F)}$$

where, PEV, $\sigma_{g}^{2}$ and F represent the prediction error variance, additive genetic variance and inbreeding coefficient of an animal.

The calculated reliability between individuals with phenotypes (heifer and cows) and sire group was compared.

In addition, genetic and phenotypic trends based on the birth year of individuals were compared by each trait and lactation using the estimated breeding values from animal model analyses.

## RESULTS

### Basic characteristics of selected reproductive traits

The dataset shows that the reproductive trait decreases as the lactation number increases (Table 1). With CF, which represents the recovery of cows, we found that cows at third lactation required about 6 more days for their recovery than that of first lactation cows. The CCR was also reduced by ~5% in the third lactation compared to the first. We also observed that the interval from the first insemination to the last insemination, a characteristic related to CCR, also increased by 7 days. The DO phenotype also increased by 12 days from first to third lactation.

**Figure 1:**
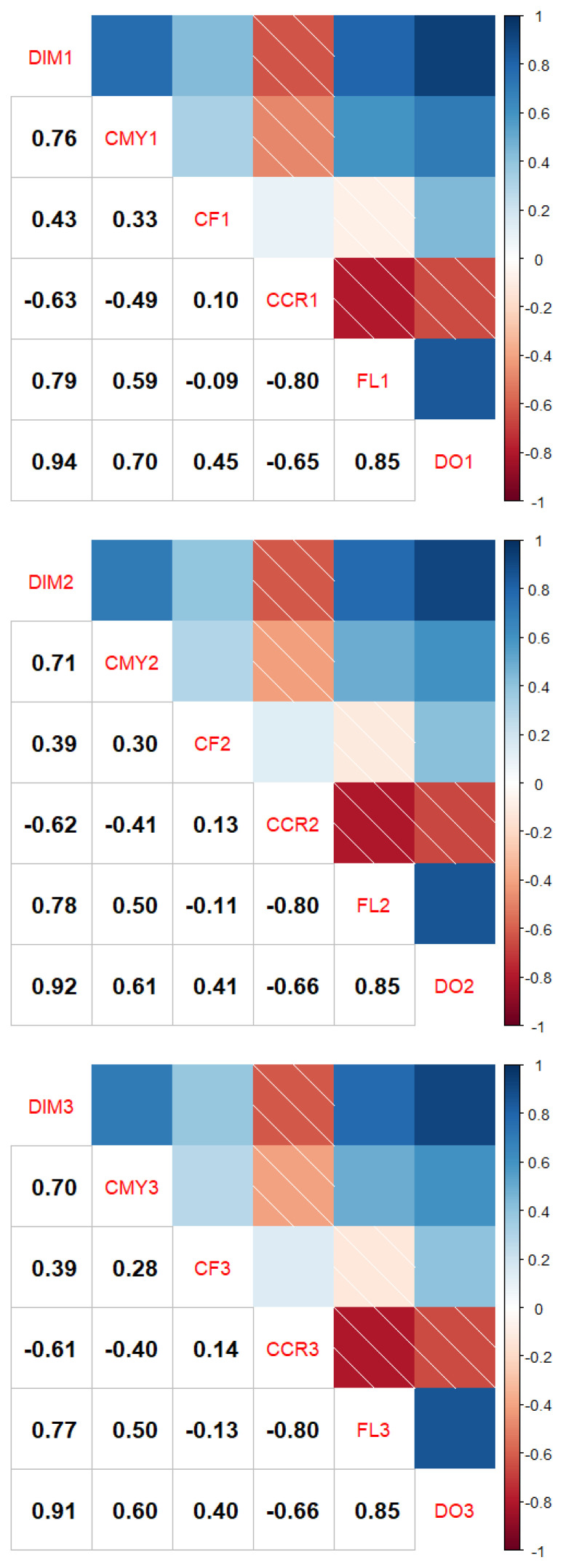
Heatmap showing correlations between total days in milk per lactation (DIM), cumulative milking yield (CMY) and reproductive traits (CF, CCR, FL, DO), by lactation (1,2,3, lactation number; (a) first lactation; (b) second lactation; (c) third lactation).

Our data also shows properties of the negative relationship between reproductive traits and productive traits. The comparison of correlation coefficient shows a negative correlation between the days in milk, the cumulative milk yield and the conception rate, and a positive correlation between the FL or DO (Figure 1). This implies that if cows are milked more and longer, the fertility decreases.

### Result of genetic parameter estimation

In this study, most genetic parameters for reproductive traits are modest. Specifically, for the HCR, the T1 trait using a single-trait model, the genetic and residual variances were estimated to be 5.33 and 509.15, respectively, resulting in a very low heritability (*h*^2^) of 0.010. As depicted in Table 2, when applying the multiple lactation model to estimate the genetic parameters of CF, CCR, FL, and DO, the T2 to T5 traits, we also noted low heritabilities across these traits. However, there was a detectable genetic correlation between lactations. The genetic correlations between the first and second lactations were notably high for most traits, except for CF. Specifically, genetic correlation estimates were 0.556 for CF, 0.890 for CCR, 0.911 for FL, and 0.871 for DO. Except for FL, the genetic correlations between the second and third lactations were also high for most traits, with 0.914 for CF, 0.489 for CCR, and 0.485 for DO (0.148 for FL). Within the scope of T2 to T5 traits, which were analyzed using multiple lactation models, heritability demonstrated a slight decreasing trend with increasing parity, except for the CF trait.

### Reliabilities of estimated breeding value for traits and their yearly trends

The theoretical reliability of estimated breeding value for reproductive traits obtained using the variance of prediction errors (PEV) for each individual and the inbreeding coefficient. For heifers and cows with phenotypic information, the average estimated reliability across most traits was very low. This outcome primarily reflects the influence of heritability on theoretical reliability, which remains significant unless there is a substantial contribution from progenies. Notably, the variation in reliability across different lactations mirrors the pattern observed in heritability values. Nevertheless, individuals with substantial progeny contributions, particularly proven sires expected to have numerous offspring, exhibited theoretical reliabilities of 0.8 or higher for specific traits. This higher reliability highlights the potential for genetic improvement through strategic sire selection despite the overall low heritability of these traits (Table 3).

**Figure 2:**
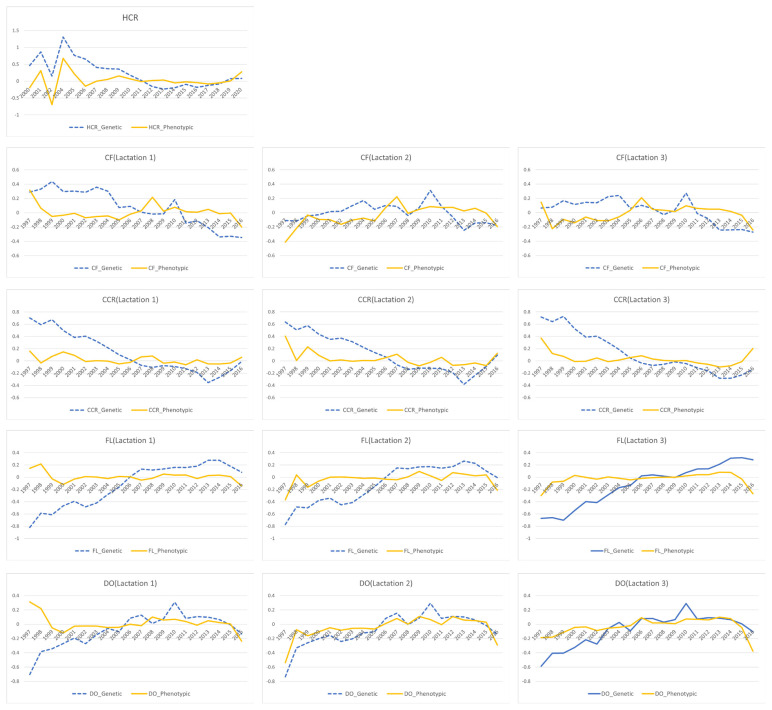
Genetic and phenotypic trends by birth year of animals for five reproductive traits in Korean Holstein. HCR, heifer conception rate; CF, interval from calving to the first insemination; CCR, cow conception rate; FL, interval from the first to the last insemination; DO, days open.

Korea has been focusing on improving productive traits for a long time, so it is expected that the genetic improvement of reproductive traits has decreased, considering the results of many studies that show negative genetic correlation between reproductive and productive traits. The yearly phenotypic and genetic changes for traits are shown in heifers and cows with phenotypes (Figure 2).

It is difficult to find trends any noticeable phenotypic trend across traits in this study. However, after closer inspection from 1997 to 2013, we find some gradual decline in the genetic trends of reproductive traits. CF continues to increase, CCR decreases, FL increases, and DO tends to increase. Contrary to this trend, it seems that genetic improvement has occurred slowly for reproductive traits since 2014. As Korean Holsteins have not been exposed to selection for reproductive traits, this trend could be an indirect response to the use of imported genetic resources.

## DISCUSSION

Similar to our study, Muuttoranta et al [18] also reported on genetic parameters for reproductive traits. We find their estimated heritability value of the HCR to be comparable (0.008) to the lower estimate (0.010) in our study. However, the CF trait exhibited slightly higher heritability values across the first to third lactations (0.057, 0.055, and 0.067, respectively) than our findings of 0.035, 0.018, and 0.031. For the FL, the former study reported heritability values of 0.041, 0.048, and 0.041 for the first to third lactations, surpassing our results of 0.025, 0.012, and 0.007. Similarly, while their CCR *h*^2^ values were noted as 0.025, 0.030, and 0.029, our study reported 0.021, 0.011, and 0.007, respectively. Otwinowska-Mindur et al [22] also estimated the genetic parameters for Polish Holstein cattle, and they generally found higher heritability values than those we observed. We also observed further support for our lower *h*^2^ of CCR from the reported values (0.010 to 0.015) on a Chinese Holstein population by Liu et al [23]. However, their *h*^2^ values of CF reported were higher, at 0.102, 0.078, and 0.069, respectively. Similarly, they showed the *h*^2^ of FL to be 0.034, 0.030, and 0.026 and those of DO to be 0.049, 0.037, and 0.039 for first three lactations, respectively, slightly higher than our values. Additionally, our low *h*^2^ for HCR also corroborates with Liu et al [23]. As for the genetic correlation, since Muuttoranta et al [18] used multiple trait and multiple lactation models, it is difficult to compare directly, but the genetic correlation among lactations in the same trait was higher or similar than in this study. Aguilar et al [19] have different models from this study and genomic data was used, but the *h*^2^ of CCR traits estimated by forming the HY contemporary group in the model was 0.018 and 0.022 and 0.026 in the third lactation model, similar to the results of this study in the first lactation and higher in the rest of the lactations than in this study, and the genetic correlation was similar to 0.877 between first and second lactation, but the genetic correlation of this study was lower in the rest of the traits combinations. In addition, the study of Aguilar et al [19] included service sire in the model with another random effect, and as a result of attempting to apply the same method in this study, our results were somewhat strange in some traits and were excluded from this study. Our genetic trend outcomes can be compared with the report on Icelandic dairy cows by Þórarinsdóttir et al [24]. Although their study did not reveal any significant trends over an extended period due to a lack of selection of those traits, our data exhibited a rebound starting in 2014. However, they only observed similar patterns in the conception rates of heifers, and the absence of data post-2016 limited further comparisons.

Overall, the estimated heritability for reproductive traits in this study is lower compared to findings from other research. Reproductive traits generally have very low heritability and exhibit minimal genetic variation in genetic parameter estimation, making them particularly susceptible to the influence of outliers that can lead to abnormal variance estimates. Eliminating potential data entry errors and recording inaccuracies from the collected data enhances accuracy and consistency, leading to more precise genetic parameter estimation. However, the exclusion of outliers which were defined as values that significantly deviate from the mean value may naturally result in a reduction of the estimated variance. Consequently, the low heritability observed in this study may be attributed to a greater reduction in genetic variance included in the model compared to the reduction in residual variance, which was not accounted for by the model in the overall dataset with reduced variance. That is, excessive data quality checks may have caused low heritability. Optimum data quality check would be very difficult. It would be general to follow international standards. This discrepancy may be addressed by the accumulation of higher-quality fertility data. Furthermore, to enhance the potential for genetic improvement considering the low heritability, it is advisable for future studies to consider more comprehensive models, such as the multiple trait-lactation polymorphic model, especially as more data becomes available.

In this study, the heritability estimates of the reproductive traits were relatively low. However, a comparison with international research suggests that the results obtained in Korea are not anomalous. In the review of research papers by Shao et al [25], it was observed that the heritability of reproductive traits is predominantly low. Despite this, the traits selected over the past century are increasingly recognized as modern improvement traits, particularly those that have been emphasized since the 2000s [26]. Observing genetic trends, it is evident that the importance of reproductive traits has been recognized globally, leading to the selection of genetically superior individuals. It appears that Korea has also been indirectly influenced by these global trends. Therefore, if Korea were to select elite breeding stock specifically for improving reproductive traits, such a strategy could potentially accelerate this trend.

Despite the very low heritability, certain proven sires demonstrated a relatively high reliability of estimated breeding values. The existence of genetic correlations across different lactations suggests that with the accumulation of extensive and high-quality data, it would be feasible to advance the improvement of dairy cattle reproductive traits in Korea. Proper data management and analysis are crucial to achieve significant genetic progress in this area.

## Figures and Tables

**Table 1:** Number of records, mean and standard deviation for five reproductive traits (HCR, CF, CCR, FL and DO) in Korean Holsteins

Trait/lactation	Number of records	Mean	Standard deviation
HCR	111,298	81.52	27.32
CF			
L1^1)^	33,100	80.45	38.18
L2^1)^	33,100	82.62	37.29
L3^1)^	33,100	86.15	38.34
CCR			
L1^1)^	33,100	70.51	31.16
L2^1)^	33,100	67.49	31.49
L3^1)^	33,100	66.72	31.62
FL			
L1^1)^	33,100	44.66	65.41
L2^1)^	33,100	49.55	66.74
L3^1)^	33,100	51.73	68.51
DO			
L1^1)^	33,100	124.20	72.57
L2^1)^	33,100	131.20	72.32
L3^1)^	33,100	136.90	73.84

HCR, heifer conception rate; CF, interval from calving to the first insemination; CCR, cow conception rate; FL, interval from the first to the last insemination; DO, days open.

1) L1, lactation 1; L2, lactation 2; L3, lactation 3.

**Table 2:** Estimated heritabilities (diagonal elements of L1 to L3), genetic correlations (upper off-diagonal elements of L1 to L3) and variance components for five reproductive traits in Korean Holsteins

Trait	Heritability			Genetic variance	Residual variance

HCR	0.010			5.33	509.15

Trait/lactation	Heritability and genetic correlation				

	L1	L2	L3		
CF					
L1^1)^	0.035	0.566	0.835	40.88	1,121.80
L2^1)^	-	0.018	0.914	20.46	1,113.00
L3^1)^	-	-	0.031	37.49	1,176.10
CCR					
L1^1)^	0.021	0.890	0.832	15.66	733.21
L2^1)^	-	0.011	0.489	9.39	810.79
L3^1)^	-	-	0.007	6.35	846.32
FL					
L1^1)^	0.025	0.911	0.543	69.46	2,758.50
L2^1)^	-	0.012	0.148	40.97	3,356.30
L3^1)^	-	-	0.007	24.72	3,771.20
DO					
L1^1)^	0.030	0.871	0.852	84.87	2,701.40
L2^1)^	-	0.020	0.485	68.52	3,381.50
L3^1)^	-	-	0.008	31.67	3,862.30

HCR, heifer conception rate; CF, interval from calving to the first insemination; CCR, cow conception rate; FL, interval from the first to the last insemination; DO, days open.

1) L1, lactation 1; L2, lactation 2; L3, lactation 3.

**Table 3:** Theoretical reliability for five reproductive traits in Korean Holsteins

Trait/lactation	Cows with records		Sires	
	
	Mean	Max	Mean	Max
HCR	0.17	0.45	0.15	0.81
CF				
L1^1)^	0.16	0.46	0.16	0.79
L2^1)^	0.15	0.43	0.15	0.74
L3^1)^	0.17	0.49	0.17	0.83
CCR				
L1^1)^	0.13	0.43	70.51	0.74
L2^1)^	0.11	0.38	67.49	0.66
L3^1)^	0.10	0.34	66.72	0.59
FL				
L1^1)^	0.13	0.43	44.66	0.75
L2^1)^	0.12	0.40	49.55	0.70
L3^1)^	0.07	0.26	51.73	0.59
DO				
L1^1)^	0.16	0.46	0.16	0.80
L2^1)^	0.14	0.43	0.14	0.73
L3^1)^	0.12	0.37	0.12	0.64

HCR, heifer conception rate; CF, interval from calving to the first insemination; CCR, cow conception rate; FL, interval from the first to the last insemination; DO, days open.

1) L1, lactation 1; L2, lactation 2; L3, lactation 3.
